# Comparative Analysis of Antioxidant Compounds and Antioxidative Properties of Thai Indigenous Rice: Effects of Rice Variety and Processing Condition

**DOI:** 10.3390/molecules27165180

**Published:** 2022-08-14

**Authors:** Pijug Summpunn, Worawan Panpipat, Supranee Manurakchinakorn, Phuangthip Bhoopong, Ling-Zhi Cheong, Manat Chaijan

**Affiliations:** 1 Food Technology and Innovation Research Center of Excellence, School of Agricultural Technology and Food Industry, Walailak University, Nakhon Si Thammarat 80160, Thailand; 2 Zhejiang-Malaysia Joint Research Laboratory for Agricultural Product Processing and Nutrition, College of Food and Pharmaceutical Science, Ningbo University, Ningbo 315211, China

**Keywords:** rice, antioxidant activity, phenolic compounds, brown rice, germination, rice grass

## Abstract

Indigenous southern Thai non-glutinous rice varieties Kaab Dum, Khai Mod Rin, Yar Ko, Yoom Noon, and Look Lai made under four different processing conditions, white rice, brown rice, germinated brown rice, and rice grass, were assessed for antioxidant components and in vitro antioxidative activities. According to the findings, rice’s antioxidant components and antioxidant activity were considerably impacted by both variety and processing. High levels of total extractable phenolic compounds (164–314 mg gallic acid equivalent (GAE)/kg, dry weight (dw)) and carotenoid (0.92–8.65 mg/100 g, dw) were found in all rice varieties, especially in rice grass and germinated brown rice, indicating that milling to generate white rice had an adverse effect on those components. Additionally, after germination, a higher γ-oryzanol concentration (9–14 mg/100 g, dw) was found. All rice varieties had higher ascorbic acid, phenolic compound, and carotenoid contents after sprouting. Overall, Yoom Noon rice grass had the highest total extractable phenolic content (*p* < 0.05). The rice grass from Yoom Noon/Look Lai/Kaab Dum had the highest ascorbic acid content (*p* < 0.05). The total carotenoid concentration of Look Lai rice grass was the highest, and Yoom Noon’s germinated brown rice had the highest γ-oryzanol content (*p* < 0.05). All rice varieties’ aqueous extracts had remarkable ABTS free radical scavenging activity, with Khai Mod Rin reaching the highest maximum value of 42.56 mmol Trolox equivalent/kg dw. Other antioxidant mechanisms, however, were quite low. Compared to germinated brown rice, brown rice, and white rice, rice grass often tended to have stronger antioxidant activity. Yar Ko rice grass was found to have the highest DPPH free radical scavenging activity (3.8 mmol Trolox equivalent/kg dw) and ferric reducing antioxidant power (FRAP) (4.6 mmol Trolox equivalent/kg dw) (*p* < 0.05). Khai Mod Rice grass had the most pronounced metal chelation activity (1.14 mmol EDTA equivalent/kg dw) (*p* < 0.05). The rice variety and processing conditions, therefore, influenced the antioxidant compounds and antioxidative properties of Thai indigenous rice. The results can be used as a guide to select the optimal rice variety and primary processing in order to satisfy the needs of farmers who want to produce rice as a functional ingredient and to promote the consumption of indigenous rice by health-conscious consumers.

## 1. Introduction

Rice (*Oryza sativa* L.) is a significant cereal crop for over half of the world’s population, particularly in Asia, because it is a major source of carbohydrates and other nutrients [1]. Because of its high consumption and important supply of carbohydrates, vitamins, minerals, and bioactive substances, rice is an excellent vehicle for delivering nutrients to these populations [2,3,4,5]. Rice is mostly consumed as intact kernels, but rice flour can be utilized in several food products, including conventional foods, noodles, baked goods, extruded items, and innovative products (such as snacks, gluten-free processed foods, and infant foods) [2,3,6]. Due to rising consumer demand, it is anticipated that worldwide rice production will double by 2050 [7].

Rice varieties differ greatly in terms of chemical, physical, thermal, and pasting properties, all of which are influenced by genetic and environmental factors [2,3,5]. Rice normally contains 70–80% carbohydrate, 6–8% protein, 1–3% fat, 0.3–1.5% ash, and 0.2–1% fiber, depending on genotype, agronomic and cultivation circumstances, storage, and processing features [1,8]. Rice grains have varying physicochemical qualities depending on the variety, and the starch quality has a significant impact on their cooking properties [9]. Thailand is one of the most important rice-producing countries in Asia. Rice is a major Thai commercial crop, and its grains are consumed as a staple food, with a wide range of rice varieties available across the country [10]. Different regions of Thailand grow traditional rice varieties because some consumers still utilize local rice as a staple diet [4,5,11,12]. In southern Thailand, particularly in the Pak Panang Basin of Nakhon Si Thammarat, many indigenous rice varieties are widely farmed. According to farmers’ interviews, the top five native rice varieties cultivated in this region are Kaab Dum, Khai Mod Rin, Yar Ko, Yoom Noon, and Look Lai.

Aside from rice variety, rice processing, such as milling, germination, and growing to rice grass, can impact the compositional, nutritional, physicochemical, and bioactive aspects of rice [1,2,3,4,5,8,10]. Due to its potential health benefits, brown rice has gained a lot of attention as an important type of whole grain. Brown rice’s health benefits have recently been highly publicized [8]. Brown rice is composed of three layers: bran, embryo, and endosperm; when brown rice is polished into white rice, the majority of the bran and embryo are removed [8]. Thus, brown rice is abundant in phytonutrients such as phenolics, dietary fiber, vitamins, minerals, γ-oryzanols, and carotenoids, which are all known to reduce the risk of many diseases [13,14]. These compounds may function as natural antioxidants to possibly prevent the destruction of biomolecules such as lipids, proteins, and DNA. [13,15]. Among these substances, polyphenols have been identified as the main active component for anti-oxidation [16,17]. In addition, whole grains’ nutritional value is greatly improved during germination and sprouting [4,10,18]. Because of their health-promoting features and high nutritional qualities, such as amino acids, fiber, trace minerals, vitamins, flavonoids, and phenolic acids, sprouts and cereal grasses have recently received increased attention as functional foods [19,20,21,22,23]. Germination is a bioprocess that boosts secondary metabolite synthesis, such as polyphenols and γ-aminobutyric acid (GABA) [18]. In cereal grasses at the jointing stage, antioxidants and phytonutrients are abundant [10,18,24]. When the rice grass is in the jointing stage, just before the second leaf appears, it can be harvested [10]. According to research on Thai rice varieties, rice grass juice, especially from the Kum Doisaket variety, demonstrated antioxidant and DNA-protective properties [10].

**Figure 1:**
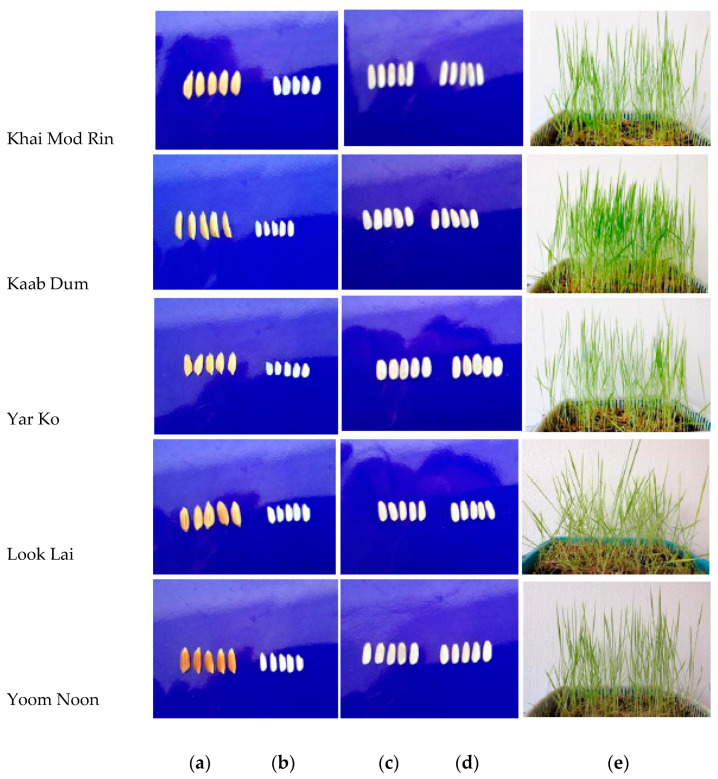
Appearances of paddy (**a**), white rice (**b**), brown rice (**c**), germinated brown rice (**d**), and rice grass (**e**) of southern Thai indigenous non-colored rice varieties, including Khai Mod Rin, Kaab Dum, Yar Ko, Look Lai, and Yoom Noon.

Recently, research has changed to develop functional food products with bioactive ingredients as awareness of nutrition and health has grown. Numerous investigations are being carried out to establish how polishing affects the functional, physicochemical, and nutritional qualities of colored and non-colored rice varieties [1,2,8,11,25]. However, the antioxidant components and properties of the non-colored rice grown in southern Thailand, as influenced by rice variety and processing conditions, have received relatively limited study [5]. Therefore, this research sought to determine the impact of rice variety and processing conditions (white rice, brown rice, germinated brown rice, and rice grass) (Figure 1) on some antioxidant substances and antioxidant activity of five native non-glutinous short grain Thai rice varieties: Khai Mod Rin, Kaab Dum, Yar Ko, Look Lai, and Yoom Noon. The findings will offer fundamental information on the antioxidant compounds and their antioxidative properties of native Thai rice, which can be applied as an alternative for healthy food preparations and functional ingredients in both domestic and commercial scenarios.

## 2. Results and Discussion

### 2.1. Total Extractable Phenolic Content

**Figure 2:**
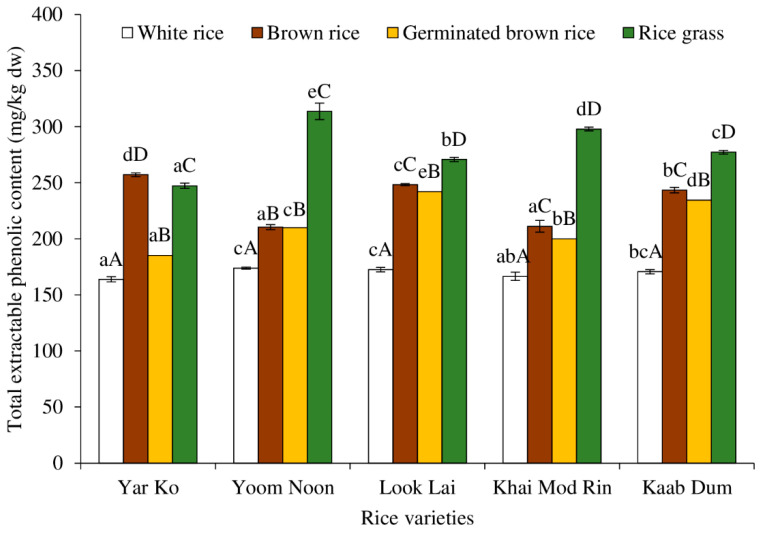
Effect of rice variety and processing condition on total extractable phenolic content of Thai indigenous rice. Bars represent the standard deviations from triplicate determinations. Different lowercase letters within the same processing condition indicate significant differences (*p* ˂ 0.05). Different uppercase letters within the same variety indicate significant differences (*p* ˂ 0.05).

Figure 2 displays the total extractable phenolic contents of five indigenous rice varieties processed using various methods. Among all rice varieties, white rice showed the lowest total extractable phenolic content (*p* < 0.05). Except for the Yar Ko variety, where the highest total extractable phenolic content was identified in brown rice, the highest total extractable phenolic content was discovered in rice grass. In Look Lai, Khai Mod Rin, and Kaab Dum, the order of total extractable phenolic content was typically rice grass > brown rice > germinated brown rice > white rice. In Yoom Noon, the difference in total extractable phenolic content between brown rice and germinated brown rice was not statistically significant (*p* > 0.05). In general, whole rice grains, which include the endosperm, embryo (or germ), and bran, provide health benefits in addition to offering nutrients. Shao et al. [26] reported the distribution of total phenolic content in the endosperm, embryo, and bran of white, red, and black rice grains. The bran typically had the highest total phenolic content (7.35 mg gallic acid equivalent (GAE)/g), contributing 60%, 86%, and 84% of the phenolics in white, red, and black rice, respectively. The average total phenolic content of the embryo and endosperm were 2.79 and 0.11 mg GAE/g, accounting for 17% and 23%, 4% and 10%, and 7% and 9% in white, red, and black rice, respectively [26]. Polyphenolics are secondary plant metabolites that are created to aid in plant growth [10,18,24]. As a result, rice grass has the highest total extractable phenolic content.

Phenolic compounds which were present in the rice bran during pre-soaking may be leached away in order to generate germinated brown rice. As a result, when compared to raw brown rice, germinated brown rice frequently had lower total phenolic content. The white rice, which has the lowest total phenolic content due to the complete removal of the rice bran, exhibits this effect noticeably. According to Tian et al. [27], the highest concentration of total extractable phenolics was discovered in brown rice, followed by germinated brown rice and white rice, in that order. When compared to parent brown rice, the number of phenolic esters such as hydroxycinnamate sucrose esters in germinated brown rice was reduced by 70% [27]. According to Goufo and Trindade [28], rice phenolic is made up of 12–28% hydroxybenzoic acids and 61–89% hydroxycinnamic acids. In southern Thai indigenous brown rice, a number of phenolic compounds and their derivatives, including phenolic acids, esters, and glucosides, were found [5]. Some indigenous brown rice from southern Thailand has been found to contain specific compounds, including benzoic acid, *m*-salicylic acid, glucocaffeic acid, 6-caffeoylsucrose, dihydroferulic acid 4-O-glucuronide, natsudaidain 3-(4-O-3-hydroxy-3-methylglutaroylglucoside), (R)-2,3-dihydro-3,5-dihydroxy-2-oxo-3-indoleacetic acid, 1-O-cinnamoyl-beta-D-gentiobiose, and 3′,6-disinapoylsucrose [5].

### 2.2. Ascorbic Acid Content

**Figure 3:**
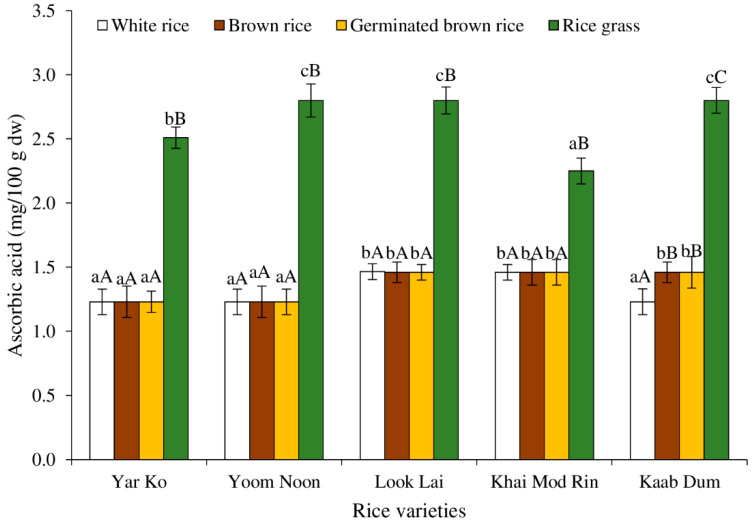
Effect of rice variety and processing condition on ascorbic acid content of Thai indigenous rice. Bars represent the standard deviations from triplicate determinations. Different lowercase letters within the same processing condition indicate significant differences (*p* ˂ 0.05). Different uppercase letters within the same variety indicate significant differences (*p* ˂ 0.05).

Figure 3 displays the ascorbic acid content of five native rice varieties processed differently. When the ascorbic content of white rice, brown rice, and germinated brown rice was compared, it was discovered that Khai Mod Rin, Kaab Dum, and Look Lai had ascorbic acid contents that were comparable to and higher than those of Yar Ko and Yoom Noon. The ascorbic acid level of all rice grass samples was higher than that of other processing conditions (*p* < 0.05). The quantities of ascorbic acid in rice grass were highest in Kaab Dum, Look Lai, and Yoom Noon, then Yar Ko and Khai Mod Rin (*p* < 0.05). According to Singh and Prasad [29], neither polished rice nor brown rice contained ascorbic acid. In this study, trace amounts of ascorbic acids (<1.5 mg/100 g dw) were detected in white rice, brown rice, and germinated brown rice using the spectrophotometric approach. However, these contents were almost negligible if they were calculated on a wet basis.

With the exception of Kaab Dum, where there was a drop in ascorbic acid level in white rice (*p* < 0.05), processing brown rice into white rice and germinated rice had no influence on the ascorbic acid content in any of the five rice samples (*p* > 0.05). Ascorbic acid, which has vitamin C activity, is a crucial antioxidant and aids in a number of physiological processes in plants, including cell division, growth, and photosynthesis [30]. Since ascorbic acid is typically present in all plant tissue and is particularly abundant in young leaves and photosynthetic tissue [31,32], the production of rice grass boosted the ascorbic acid level in all five rice varieties. The results were consistent with the report of Liskko et al. [33], which found that ascorbic acid increased when the rice germinated at the early vegetative stage.

### 2.3. Carotenoid Content

**Figure 4:**
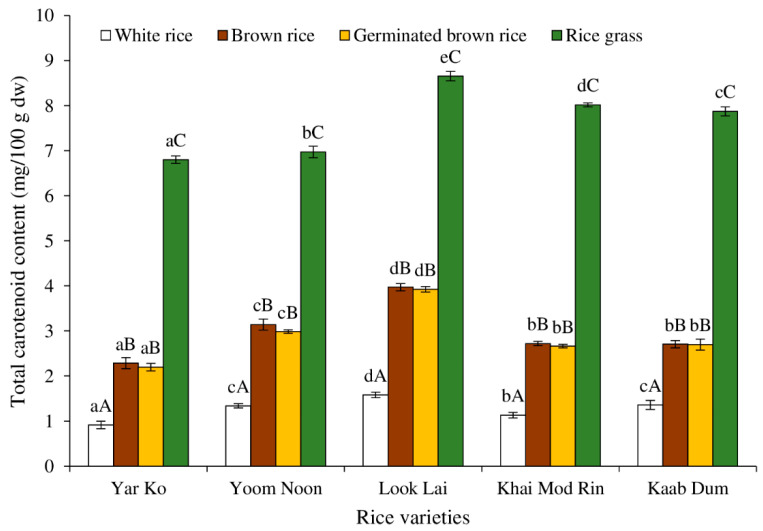
Effect of rice variety and processing condition on total carotenoid content of Thai indigenous rice. Bars represent the standard deviations from triplicate determinations. Different lowercase letters within the same processing condition indicate significant differences (*p* ˂ 0.05). Different uppercase letters within the same variety indicate significant differences (*p* ˂ 0.05).

Figure 4 depicts the carotenoid content of five indigenous rice varieties processed differently. The variation in rice’s carotenoid content was dependent on the varieties and processing techniques used. However, the rice varieties had less of an impact on the carotenoid content than the processing method. Rice grass had the highest level of carotenoid content, while white rice had the lowest level (*p* < 0.05). Brown rice and germinated brown rice both contained moderate amounts of carotenoids. Because chlorophyll obscures their appearance, the carotenoid pigments found in plant leaves are not readily visible (green). The carotenoids’ prominent colors are yellow, orange, and red when the chlorophyll is absent. Therefore, despite the presence of carotenoids, young leaves are green [34,35].

Rice grass had the highest concentration of carotenoids, followed by brown rice, germinated brown rice, and white rice. This proved that carotenoids were lost during the polishing of rice grains. This outcome was in line with Lamberts and Delcour’s report [36], which discovered that the exterior layers of rice grains had a significant amount of red and yellow pigments but that the interior seed did not.

Notably, all five rice tests showed that the carotenoids in brown rice and germinated brown rice were identical. Because carotenoids are not generally water soluble, very little carotenoid was lost throughout the soaking and washing steps of making germinated brown rice. The carotenoid levels of the five rice samples connected to grain processing varied depending on the rice varieties’ input. The white rice with the largest amount of carotenoids was Look Lai, followed by Kaab Dum/Yoom Noon, Khai Mod Rin, and Yar Ko.

With regard to brown rice and germinated brown rice, Look Lai had the highest carotenoid content, followed by Yoom Noon, Khai Mod Rin/Kaab Dum, and Yar Ko (*p* < 0.05). The carotenoid level in rice grass was highest in Look Lai, followed by Khai Mod Rin, Kaab Dum, Yoom Noon, and Yar Ko, in that order (*p* < 0.05). Interesting to note is that Look Lai in this study showed a greater carotenoid content than the other examined varieties. In brown and milled rice from 39 aromatic rice varieties, beta-carotene levels were reported by Renuka et al. [37] in 2016. Beta-carotene content was discovered to range from 0.008 to 0.2 mg/100 g in milled rice and from 0.11 to 0.21 mg/100 g in brown rice. In comparison to that report, all five native rice varieties had greater carotenoid contents. It should be mentioned that Renuka et al. [37] reported the beta-carotene contents, whereas, in our investigation, we provided the total carotenoid levels.

### 2.4. γ-Oryzanol Content

**Figure 5:**
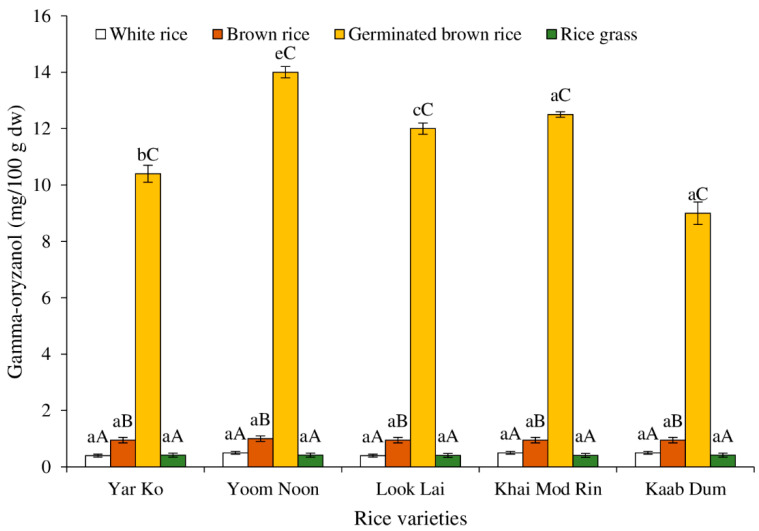
Effect of rice variety and processing condition on γ-oryzanol content of Thai indigenous rice. Bars represent the standard deviations from triplicate determinations. Different lowercase letters within the same processing condition indicate significant differences (*p* ˂ 0.05). Different uppercase letters within the same variety indicate significant differences (*p* ˂ 0.05).

Figure 5 illustrates the levels of γ-oryzanol in five indigenous rice varieties produced using various techniques. All five rice samples’ levels of γ-oryzanol were comparable between white and brown rice (*p* > 0.05). When compared to brown rice with different levels across rice varieties, the concentration of γ-oryzanol increases in brown rice that has germinated by 11.5 to 16 times. The germinated brown rice variety with the highest concentration of γ-oryzanol was Yoom Noon, followed by Khai Mod Rin, Look Lai, Yar Ko, and Kaab Dum, respectively (*p* < 0.05). When compared to brown rice, the level of γ-oryzanol was reduced in the rice grass of Khai Mod Rin, Look Lai, and Yoom Noon but not Kaab Dum and Yar Ko. Rice bran oil contains significant levels of γ-oryzanol. γ-oryzanol is a nutritional substance found in large amounts in rice bran oil. The nutritional function of γ-oryzanol involves antioxidant properties, lowering blood cholesterol and triglyceride levels, increasing high-density lipoprotein, decreasing the amount of sugar in blood circulation, and increasing the level of insulin in diabetes patients [38,39,40]. Several biochemical processes were altered, and bioactive substances, such as γ-oryzanol, were generated during the germination phase [41]. All five samples of brown rice used in this investigation showed an increase in the level of γ-oryzanol during germination. These findings were in line with the findings of Cáceres et al. [42], who discovered that when cultivated at 34 °C for 48 h, the accumulation of γ-oryzanol in the germinated brown rice of Indica SLF09 increased from 11.17 mg/100 g (dw) to 16.75 mg/100 g (dw).

### 2.5. DPPH^●^ Scavenging Activity

**Figure 6:**
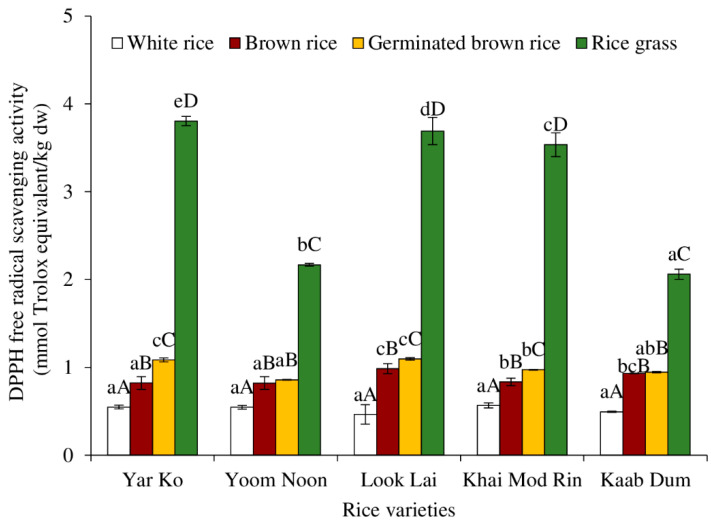
Effect of rice variety and processing condition on DPPH free radical scavenging activity of aqueous extract of Thai indigenous rice. Bars represent the standard deviations from triplicate determinations. Different lowercase letters within the same processing condition indicate significant differences (*p* ˂ 0.05). Different uppercase letters within the same variety indicate significant differences (*p* ˂ 0.05).

Figure 6 displays the DPPH^●^ scavenging capabilities of native rice extracts treated to various procedures. Various phytochemical components of rice have a variety of antioxidant activities, which can be linked to their potential health advantages [38]. Rice is considered a rich source of antioxidant molecules such as vitamin E, γ-oryzanol, phenolic compounds, anthocyanin and proanthocyanidin, flavonoids, carotenoids, and phytosterols [38]. The highest DPPH scavenging activity was found in rice grass of all rice varieties, followed by germinated brown rice, brown rice, and white rice, respectively (*p* < 0.05). Several antioxidants were present in rice grass, especially total extractable phenolic compounds (Figure 2), ascorbic acid (Figure 3), and total carotenoids (Figure 4). Additionally, it is possible that the antioxidant power was brought on by the presence of chlorophyll, which has been linked to antioxidant activity [43].

A good association between phenolic substances and DPPH^●^ inhibition, which stands for radical inhibition, was found by Shao et al. [26] and Moniruzzaman et al. [44]. However, factors such as molecular structure, position and number of hydroxyl groups, polarity, and—most significantly—the bond dissociation energy needed to separate the hydrogen atom from the compound affect their antioxidative activity [45,46]. Yar Ko rice grass showed the highest DPPH^●^ scavenging activity when the impact of rice type was taken into account, followed by those from Look Lai, Khai Mod Rin, Yoom Noon, and Kaab Dum, respectively (*p* < 0.05). For germinated brown rice, the DPPH^●^ scavenging activity of Look Lai ≥ Yar Ko > Khai Mod Rin ≥ Kaab Dum ≥ Yoom Noon. For brown rice, the DPPH^●^ scavenging activity of Look Lai ≥ Kaab Dum ≥ Khai Mod Rin ≥ Yoom Noon = Yar Ko. Because of the milling and polishing processes, which deplete antioxidant components, the DPPH scavenging activities of white rice were not varied across five varieties (*p* > 0.05). The degree of milling is a significant aspect that can affect the nutritional value and antioxidant activity of rice [47]. The quantity of the antioxidant molecule quercetin, ferulic, and coumaric acids decreased as the degree of milling of japonica and indica brown rice increased [47].

### 2.6. ABTS^●+^ Scavenging Activity

**Figure 7:**
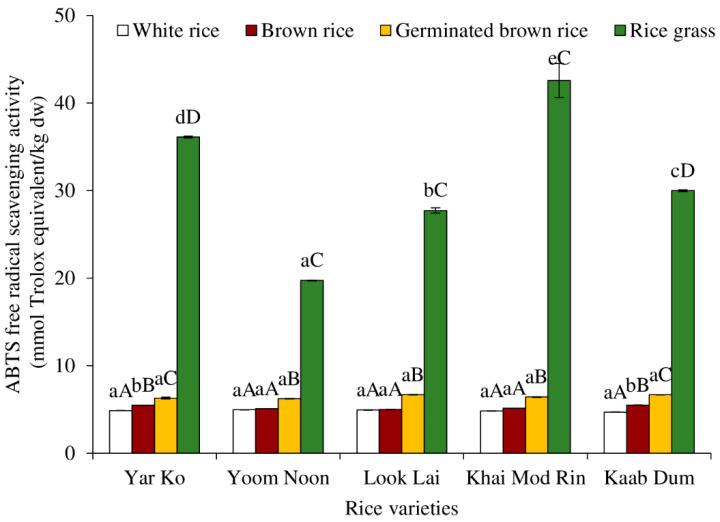
Effect of rice variety and processing condition on ABTS free radical scavenging activity of aqueous extract of Thai indigenous rice. Bars represent the standard deviations from triplicate determinations. Different lowercase letters within the same processing condition indicate significant differences (*p* ˂ 0.05). Different uppercase letters within the same variety indicate significant differences (*p* ˂ 0.05).

Figure 7 displays the ABTS^●+^ scavenging activities of indigenous rice extracts that were treated to various procedures. Rice grass displayed the highest ABTS^●+^ scavenging activity among all rice varieties, followed by brown rice that had been germinated, brown rice, and white rice, in that order (*p* < 0.05), as found in the DPPH^●^ scavenging activity (Figure 6). The ABTS^●+^ scavenging activity (~5–40 mmol Trolox equivalent/kg dw) was almost 10 times greater than the DPPH^●^ scavenging activity (~0.5–4 mmol Trolox equivalent/kg dw). This was because the water-soluble active compounds (such as water-soluble phenolics and ascorbic acid) could be extracted, which led to higher scavenging activity against the water-soluble free radical, ABTS^●+^, than the hydrophobic free radical, DPPH.

Regarding the rice variety, rice grass of Khai Mod Rin had the highest ABTS scavenging activity, followed by those of Yar Ko, Kaab Dum, Look Lai, and Yoom Noon, respectively (*p* < 0.05). All types of rice demonstrated the same ABTS^●+^ scavenging activity for germinated brown rice, brown rice, and white rice (*p* > 0.05). Normally, germinated brown rice had higher ABTS^●+^ scavenging activity than brown rice and white rice (*p* < 0.05), which was due to the fact that milling and polishing can remove antioxidative compounds of rice [8]. Germination and sprouting can produce some antioxidants [4,10,18], such as phenolic compounds (Figure 2), ascorbic acid (Figure 3), and carotenoids (Figure 4).

### 2.7. FRAP

**Figure 8:**
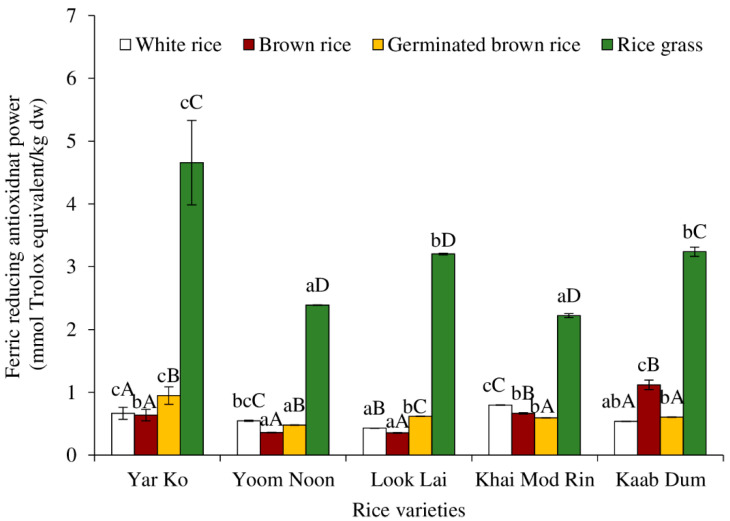
Effect of rice variety and processing condition on ferric reducing antioxidant power (FRAP) of aqueous extract of Thai indigenous rice. Bars represent the standard deviations from triplicate determinations. Different lowercase letters within the same processing condition indicate significant differences (*p* ˂ 0.05). Different uppercase letters within the same variety indicate significant differences (*p* ˂ 0.05).

The FRAP of aqueous extracts of indigenous rice subjected to different processes is depicted in Figure 8. Rice grass exhibited the highest FRAP, regardless of the type of rice (*p* < 0.05). Khanthapok et al. [10] reported that the FRAP of rice grass juice varied depending on the variety. According to one theory, phenolic compounds serve as reducing equivalents by accepting electrons from free radicals, interacting with them to create more stable products, and stopping the radical chain reaction [48]. Yar Ko had the highest FRAP among the rice grasses (*p* < 0.05), followed by Look Lai/Kaab Dum, and Khai Mod Rin/Yoom Noon, respectively. Depending on the rice variety, the FRAP values of brown rice, germinated brown rice, and white rice differed. For germinated brown rice, Yar Ko had the highest FRAP value (*p* < 0.05), while all the other varieties had the same FRAP value (*p* > 0.05). The greatest FRAP value for brown rice was recorded for Kaab Dum (*p* < 0.05), followed by Khai Mod Rin, Yar Ko, Yoom Noon, and Look Lai, respectively. For white rice, Yoom Noon, Kaab Dum, and Look Lai often had the greatest FRAP values, followed by Khai Mod Rin and Yar Ko. Based on the findings, sprouting significantly improved the FRAP of rice extract. This implied that rice sprouting produced reducing equivalents (i.e., phenolic compounds).

### 2.8. Metal Chelation

**Figure 9:**
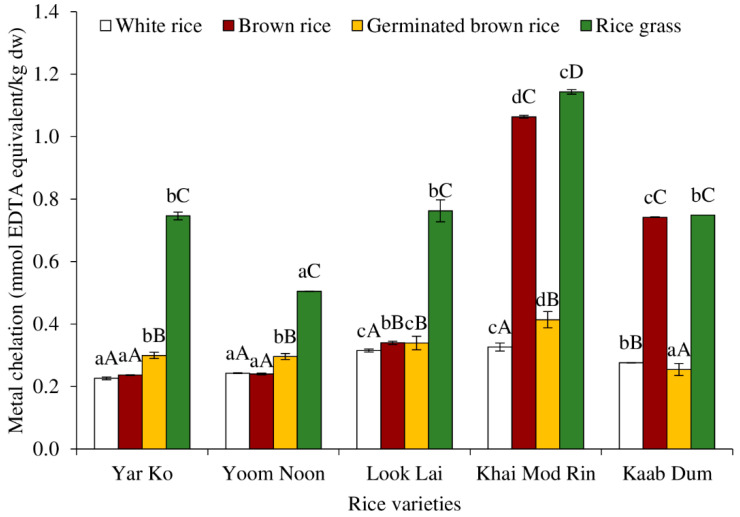
Effect of rice variety and processing condition on metal chelation of aqueous extract of Thai indigenous rice. Bars represent the standard deviations from triplicate determinations. Different lowercase letters within the same processing condition indicate significant differences (*p* ˂ 0.05). Different uppercase letters within the same variety indicate significant differences (*p* ˂ 0.05).

Metal chelation of aqueous extracts of indigenous rice subjected to different processes is shown in Figure 9. In comparison to other treatments, aqueous extracts of rice grass from all rice varieties tended to have the highest metal chelation activity (*p* < 0.05). The maximum metal chelation activity was found in the Khai Mod Rin rice grass aqueous extract, which was followed by Yar Ko/Look Lai/Kaab Dum, and Yoom Noon. According to reports, the different antioxidants present in rice grass include a lipid peroxidation inhibitor, a metal ion chelator, and a free radical scavenger [10,49]. Fascinatingly, aqueous extracts of brown rice from Khai Mod Rin and Kaab Dum tended to exhibit metal chelation comparable to or even closer to rice grass. This was most likely caused by the presence of chelating substances in brown rice of certain varieties, including phenolic compounds, carotenoids, phytic acid, and other substances [3,4,5,8]. The loss of chelating compounds through leaching and/or chemical changes during soaking and germination may have been the reason why the metal chelation was reduced when the germination was applied. After germination, the metal chelation rose to a considerable level in Yar Ko, Yoom Noon, and Look Lai. Aqueous extract of white rice from all kinds had the lowest metal chelation (*p* < 0.05), which was similar to other antioxidant mechanisms. This was likely because the chelating agent was removed during milling and polishing [8].

## 3. Materials and Methods

### 3.1. Chemicals

All chemicals used for analyses were purchased from Sigma-Aldrich Corp. (St. Louis, MO, USA).

### 3.2. Rice Samples

Five native southern Thai non-glutinous rice varieties (*Oryza sativa* L.), including Khai Mod Rin, Kaab Dum, Yar Ko, Look Lai, and Yoom Noon (12% moisture), were harvested at Pak Phanang, Nakhon Si Thammarat, Thailand (Figure 1). All of the rice employed in this study was of the non-colored variety. Utilizing a domestic method, white and brown rice were analyzed in comparison to germinated brown rice and rice grass (Figure 1). Prior to being employed in the experiment, the paddies were checked for their ability to germinate (at least 90% germination). For the preparation of germinated brown rice, brown rice was soaked in the dark for 96 h at 35 °C in water with a pH of 5 while changing the water every 6 h [4]. The Khanthapok et al. [10] method was modified for the preparation of rice grass. Rice paddies were cleaned and left to soak in tap water all night. Seeds were placed in vermiculite medium in plastic trays and then irrigated with tap water until the seeds sprouted. Rice grass was cultivated using tap water and fluorescent lighting (16/8 photoperiod) at room temperature (27–29 °C). Fresh grasses were cut above ground at the jointing stage just before the second leaf appeared (Day 9), weighed, washed thrice with tap water, then distilled water, towel-dried, and used for subsequent experiments.

### 3.3. Determination of Total Extractable Phenolic Content

The total extractable phenolic content was determined by the Folin-Ciocâlteu colorimetric method [5,50,51]. The sample (0.5 g) was treated twice with 80% aqueous methanol (8 mL) in a 40 kHz-ultrasonic bath (Ultrasons-H model 3000841 JP Selecta, Barcelona, Spain) at 35 °C for 1 h. The free phenolic fraction was obtained after centrifugation at 5000× *g* (RC-5B plus centrifuge, Sorvall, Norwalk, CT, USA) for 25 min. The pH of the supernatant was then adjusted to 4.5–5.5 using 6 M HCl. DI water (20 mL) was used to wash the aforementioned residue once more. Following water removal, the samples were twice blended with 20 mL of 4 M NaOH for 2 h in an ultrasonic bath. The pH of the mixture was raised to 4.5–5.5 using 6 M HCl. The bound phenolic fraction supernatant was obtained following centrifugation under the same condition. After that, the total extractable phenolic content of the mixed fractions was determined. Briefly, 100 μL of the sample was mixed with 2.0 mL Folin-Ciocâlteu reagent (previously diluted to 10-fold with deionized water) and thoroughly mixed. After standing for 5 min, 15% sodium carbonate solution (1.0 mL) was added. The correspondence solution was kept in the dark for 60 min. The absorbance was read at 765 nm using a Libra S22 UV-Visible spectrophotometer (Biochrom, Cambridge, England). The amount of GAE per amount of dried material represents the total extractable phenolic content.

### 3.4. Determination of Ascorbic Acid Content

The total ascorbic acid content was measured using the AOAC [52]. In a nutshell, for extraction, sample (1 g) was macerated for 3 min in 10 mL of ice-cold, 5% (*w*/*v*) metaphosphoric acid produced in 10% (*w*/*v*) acetic acid. For 10 min, the extracts were centrifuged at 7000× *g*. The supernatants were filtered through Whatman No. 1 filter paper, and a volume of 25 mL was produced. Using a 2 mL sample, 2,6-dichlorophenol indophenols (0.02% *w*/*v*), thiourea (2% *w*/*v*), and 2,4-dinitrophenylhydrazine (2% in 4.5 M H_2_SO_4_) were added one at a time, and the mixture was then incubated at 50 °C for 70 min with frequent stirring. Samples were moved right away and kept in an ice bath. After adding 4 mL of the 85% H_2_SO_4_, the mixture was gently shaken to combine the contents before being left to stand for 30 min. To ascertain the total ascorbic acid content in the samples under investigation, the absorbance at 520 nm was measured and compared with a calibration curve, which was constructed using various amounts of standard ascorbic acid.

### 3.5. Determination of Total Carotenoid Content

Ground sample (2–8 g) was extracted with acetone using an IKA^®^ homogenizer (Model T25 digital Ultra-Turrax^®^, Staufen, Germany) for 1 min at 21,500 rpm. To get rid of any acetone residue, the extracts were transferred to petroleum ether and then rinsed with water. With an equivalent volume of 10% methanolic KOH, saponification was carried out in the dark at room temperature for 16–20 h. Diethyl ether and acetone were used for the recovery and washing of the saponified carotenoids. Petroleum ether was used to dilute the obtained saponified extracts to a level of 25 mL. The extinction coefficient for mixes of carotenoids (2500) and the absorbance value measured at 450 nm were used to compute the total carotenoid concentration [53].

### 3.6. Determination of γ-Oryzanol Content

A total of 1 g of pulverized sample and 5 mL of distilled water were combined with 0.2 g of ascorbic acid, and the mixture was reacted at room temperature for 30 min. The extract was added with 5 mL of a 1:1 hexane:isopropanol solution before being incubated for 30 min (room temperature). Using a syringe filter (0.45 µm), the solvent and rice piece residue were separated. The remainder of the rice piece was then extracted once more, making a total of four extractions. The four extracted solutions were combined and centrifuged at 6000× *g* for 15 min. To obtain crude samples, the organic solvent layer was separated, and the solvent was further evaporated in a rotary evaporator (60 °C). Using 2 mL of isopropanol to dissolve about 0.1 g of the crude samples, it was then analyzed at 326 nm. The pure γ-oryzanol calibration curve was used to determine the quantity of γ-oryzanol [54,55].

### 3.7. Aqueous Rice Extract Preparation and Evaluation of Antioxidant Activities

White rice, brown rice, germinated brown rice, or rice grass was mashed with a pestle in a clean mortar containing 10 vol of distilled water. The mixtures were then placed in an ultrasonic bath operating at 40 kHz and 60 °C for 2 h. Aqueous rice extracts—supernatants—were obtained after centrifugation at 10,000× *g* (20 min/4 °C) and were utilized to ascertain the antioxidant properties.

According to Sungpud et al. [56] and Chaijan and Panpipat [4], the DPPH^•^ and ABTS^•+^ scavenging activities and the ferric reducing antioxidant power (FRAP) were evaluated. Trolox (0–1 mM) was utilized as a standard for the DPPH^•^, ABTS^•+^, and FRAP assays, and the result was reported as mmole Trolox equivalents (TE)/g dried sample. Metal chelation was assessed and represented as mmol EDTA equivalent/g dried sample, according to Limsuwanmanee et al. [57].

### 3.8. Statistical Analysis

All of the tests were carried out in triplicate (n = 3). ANOVA analysis was performed on the data. The means were contrasted using Duncan’s multiple range analysis. SPSS 23.0 was used to conduct the statistical analysis (SPSS Inc., Chicago, IL, USA).

## 4. Conclusions

Thai native rice’s antioxidant components and antioxidative activities were impacted by the rice variety and processing conditions. Depending on the rice variety, processing into brown rice, germinated brown rice, or rice grass tended to improve both antioxidant components and antioxidant power. In contrast, processing into white rice resulted in a decrease in antioxidant compounds such as total extractable phenolic, ascorbic acid, total carotenoid, and γ-oryzanol, as well as antioxidant activity. In order to meet the needs of farmers who aim to produce rice as a functional ingredient and to encourage the consumption of indigenous rice by health-conscious customers, the results can be used as a reference to choose the best rice variety and primary processing.

## Data Availability

Not applicable.

## Sample Availability

Samples of the compounds are available from the authors.
